# Trained Immunity, BCG and SARS-CoV-2 General Outline and Possible Management in COVID-19

**DOI:** 10.3390/ijms24043218

**Published:** 2023-02-06

**Authors:** Paweł Zapolnik, Wojciech Kmiecik, Artur Mazur, Hanna Czajka

**Affiliations:** 1 College of Medical Sciences, University of Rzeszów, 35-315 Rzeszów, Poland; 2 St. Louis Provincial Specialist Children’s Hospital, 31-503 Kraków, Poland

**Keywords:** BCG, vaccines, innate immunity, COVID-19, SARS-CoV-2

## Abstract

The Bacillus Calmette–Guérin (BCG) vaccine has been in use for over 100 years. It protects against severe, blood-borne forms of tuberculosis. Observations indicate that it also increases immunity against other diseases. The mechanism responsible for this is trained immunity, an increased response of non-specific immune cells in repeated contact with a pathogen, not necessarily of the same species. In the following review, we present the current state of knowledge on the molecular mechanisms responsible for this process. We also seek to identify the challenges facing science in this area and consider the application of this phenomenon in managing the severe acute respiratory syndrome coronavirus 2 (SARS-CoV-2) pandemic.

## 1. Introduction–Bacillus Calmette–Guérin

Bacillus Calmette–Guérin (BCG) is a vaccine based on a live, attenuated strain of *Mycobacterium bovis* obtained by a repeated passage in culture. The vaccine was first produced in 1921 at the Pasteur Institute in Paris [1,2,3]. It is still in use today, and over the years there have been evolutionary changes in the genome of the vaccine strains that distinguish them from the original BCG used in the early 20th century [3]. Tuberculosis (TB), prior to the severe acute respiratory syndrome coronavirus 2 (SARS-CoV-2) pandemic, was the leading cause of death from a single infectious agent, and the World Health Organization (WHO) continues to recommend the use of BCG vaccines in countries with increased rates of TB [4,5]. It has been reported in studies of various populations and infectious diseases that vaccinated populations have reduced early-life mortality and fewer cases of the disease [6,7,8,9,10]. This phenomenon is probably related to the specific effect of BCG’s stimulation of non-specific immunity. Thus, subsequent contact with an infectious agent (not necessarily the same one) results in an enhanced response, independent of adaptive immunity. This process is called ‘trained immunity’. In the following article, we aim to present the state of current knowledge on the molecular mechanisms responsible for this phenomenon and the influence of BCG vaccination on its induction. We would also like to highlight the possible significance of trained immunity in the clinical course of SARS-CoV-2 infection and discuss the challenges still faced by the scientific world in this area.

## 2. Trained Immunity in General

### 2.1. Early Observations of BCG’s Effect on Innate Immunity

The first observations of the phenomenon of generating a non-specific response through the BCG vaccine were made as early as 1927. The Swedish physician Carl Näslund observed a threefold reduction in mortality among children who received BCG compared with the unvaccinated population, which was approximately 10%. Interestingly, the most significant decrease was observed in the infant group, where TB was not the predominant cause of death [11]. In 1931, vaccine co-developer Albert Calmette made a similar observation [12]. Subsequent studies in the United States and the United Kingdom reported a 25% reduction in mortality from causes other than TB in adolescents and younger children [11]. Studies conducted in Africa linked the presence of a BCG vaccination scar to reduced child mortality from febrile illnesses and malaria. In addition, no such relationship has been shown for diphtheria, tetanus, and pertussis (DTP) vaccination [6,13].

### 2.2. Innate Immunity, Immune Responses to BCG and Trained Immunity

As the first line of defence against a pathogen, innate immunity provides the initial non-specific immune response. Innate immune cells include monocytes, macrophages, dendritic cells and other granulocytes (neutrophils, eosinophils and basophils). Some authors suggest that smooth muscle and endothelial cells may also function as innate immune cells under certain conditions [14,15,16]. The innate cells recognise specific pathogen-derived molecules (pathogen-associated molecular patterns (PAMPs)) or endogenous molecules released by tissues under stress or damage factors (danger-associated molecular patterns (DAMPs)) [14]. To detect them, immune cells use pattern recognition receptors (PRRs), which include five known receptor groups: Toll-like receptors (TLRs), C-type lectin receptors (CLRs), nucleotide-binding oligomerisation domain-like receptors (NOD-like receptors, NLRs), retinoic acid-inducible gene-I-like receptors, (RIG-I-like receptors, RLRs), as well as cytoplasmic sensors such as cyclic Guanosine monophosphate-Adenosine monophosphate synthase (cyclic GMP-AMP synthase). NLR and RLR receptors are located in the cytoplasm, while TLR and CLR are located on the surface of the cell membrane or endosome membranes [17]. Once the matching molecule is recognised, cell activation and effector responses occur at several levels. The production of cytokines and chemokines that activate other cells begins, as well as phagocytosis and direct elimination of the pathogen. Antigen-presenting cells (APCs) give a start to the stimulation of the specific response, and epigenetic and metabolic reprogramming leads to the inflammatory response and the process of trained immunity [14].

The first immune cells contacting BCG are neutrophils, macrophages and dendritic cells (DCs) located at the site of vaccine administration. The immune cells recognise pathogen-associated molecular patterns, which are found in the bacterial cell wall. These include, for example, peptidoglycan [18]. It has been shown that proteins secreted by mycobacteria can act agonistically to TLR receptors, triggering macrophage stimulation [19]. One of the antigens present in the BCG cell wall, the antigen 85, leads to stimulation of the secretion of pro-inflammatory cytokines such as tumour necrosis factor α (TNF-α), interleukin 1-β (IL-1β), or interleukin 6 (IL-6). This generates further recruiting and activation of immune cells [20]. The stage of specific immunity begins when antigen-presenting cells enter nearby lymph nodes or the spleen and activate T cells via major histocompatibility complex (MHC) molecules. As a result of the further development of the immune response, CD4+ T lymphocytes and CD8+ T lymphocytes are activated. There is also induction of B lymphocytes, which begin to produce class G (IgG) immunoglobulin and which generate memory B cells [20,21]. In the case of re-exposure to *Mycobacterium tuberculosis*, an intense and specific response will be developed to protect the patient from infection and, in particular, from severe, blood-borne forms of tuberculosis.

However, the ‘trained immunity’ concept concerns innate immunity and is independent of T and B lymphocytes. In the literature, there have been descriptions of the phenomenon of enhanced non-specific response to infection after prior exposure to a microbial agent in plants and invertebrates [22,23]. Analyses investigating this phenomenon in laboratory mouse models have also been performed [24]. The term ‘trained immunity’ itself was first used by Mihai G. Netea et al. in 2011 [25]. The first study performed with humans was based on vaccinating 20 volunteers with the BCG vaccine and analysing the material collected from them before, two weeks, and three months after vaccination [26]. The authors showed increased levels of interferon γ (IFN-γ) 2 weeks after vaccination, but interestingly, significantly increased production of IFN-γ was also demonstrated after exposure of the participants’ cells to other pathogens, *Candida albicans* and *Staphylococcus aureus*. In addition, the production of pro-inflammatory cytokines (TNF-α and IL-1β) was increased after stimulation of the cells with microbial agents, which persisted even three months after vaccination. The cytokine changes were associated with a shift in the phenotype of monocytes. The proportion of cells expressing CD14, CD11b and TLR4 increased after vaccination. The authors further conducted a study in a mouse model of severe combined immunodeficiency (SCID) with deficient T and B lymphocytes, administered with BCG vaccine or normal saline solution. The aim was to prove the action of the non-specific immunity mechanism. After injection, mice were administered a lethal dose of *C. albicans*, after which survival rates were assessed to be significantly higher in the BCG-vaccinated group. With SCID in the absence of B cells and T cells, the better immune response after vaccination must have been mediated by innate immune cells, including monocytes [26]. In addition, the authors demonstrated epigenetic changes in monocytes that underlie the phenomenon of trained immunity. Subsequent studies have uncovered other pathways associated with this process. The molecular mechanisms responsible for developing trained immunity are outlined in the following section.

### 2.3. The Molecular Basis of Trained Immunity

The next step in exploring the process of trained immunity was to identify the signal transduction pathways in the cell mediating this phenomenon. There are many pathways affecting the cell’s reprogramming in this direction, and the conducted research has revealed many specific molecules important in the above process.

Kleinnijenhuis et al., in their study, as one of the first, investigated what is probably the primary mechanism responsible for the process of trained immunity, namely, epigenetic changes [26]. These involve the regulation of gene expression without a direct change in the deoxyribonucleic acid (DNA) sequence and include modifications to the DNA itself or chromatin and histones. These modifications include acetylation, methylation, ubiquitination, phosphorylation, sumoylation, adenosine diphosphate (ADP) ribosylation, deimination or proline isomerisation [27,28,29]. Kleinnijenhuis et al. [26] demonstrated increased trimethylation of histone H3 at the lysine K4 (H3K4) position for promoters of pro-inflammatory cytokines and TLR4. The application of a histone methylation inhibitor (5′-deoxy-5′-methylthio-adenosine) resulted in an almost complete reversal of BCG-generated trained immunity.

Cheng et al. [30] conducted a study on cultured human monocytes by stimulating them with β-glucan, a major component of the fungal cell wall of *Candida*. The authors observed a transformation in the metabolic characteristics of the cells from oxidative phosphorylation to glycolysis, as evidenced by increased glucose consumption, production of lactate metabolites and the ratio of oxidised and reduced forms of nicotinamide adenine dinucleotide (NAD+/NADH) in the monocytes. The observed phenomenon can be described as the Warburg effect, known for almost 100 years. It is the use of glucose in cells undergoing rapid proliferation, e.g., cancer cells or lymphocytes [31]. Evaluation of the epigenetic profile of stimulated monocytes revealed a specific methylation and acetylation pattern of the promoters of glycolysis pathway enzyme genes, as well as the mammalian target of the rapamycin (mTOR) gene. mTOR is a conserved serine/threonine kinase that regulates numerous cellular processes, including metabolism, growth, proliferation or autophagy. The kinase functions as two protein complexes, mTOR complex 1 (mTORC1) and mTOR complex 2 (mTORC2), each with distinct functions [32]. Assessment of messenger ribonucleic acid (mRNA) expression showed an up-regulation for the mTOR protein gene and the transcription factor Hypoxia-inducible factor 1 α (HIF1α), an activator of the mTOR pathway. The mTOR activation pathway appeared to be the Phosphoinositide 3-kinase/protein kinase B (PI3K/Akt) pathway, as β-glucan led to significant Akt phosphorylation. The effects of mTOR inhibitors (rapamycin) and HIF1α (ascorbate) were also analysed, demonstrating a dose-dependent inhibition of the trained immunity effect. In addition, the authors used metformin, a commonly used drug for type II diabetes, as a different mTOR inhibitor, functioning via AMP-activated protein kinase (AMPK) activation. Under in vitro conditions, metformin inhibited the trained immunity generated by β-glucan. The authors also conducted an in vivo study by administering metformin to mice that had previously been infected with a low dose of *C. albicans*. After the infection developed, survival decreased significantly, indicating the importance of the mTOR pathway in the process of trained immunity [30].

In another study, Arts et al. [33] observed a similar effect of initiating glycolysis in incubated monocytes, in this case, after BCG stimulation. Interestingly, however, the classic Warburg effect was not noted in this case, but the interaction of glycolysis with oxidative phosphorylation was. As in the previous study, the authors investigated the PI3K/Akt-mTOR pathway, demonstrating increased Akt phosphorylation after BCG exposure and an inhibitory effect on the trained immunity after treatment with inhibitors (wortmannin, rapamycin). In addition, the authors detected increased intracellular concentrations of glutamate and malate (without an increase in fumarate), which may originate from glutamine metabolism. The use of Bis-2-(5-phenylacetamido-1,3,4-thiadiazol-2-yl)ethyl sulfide (BPTES), a known inhibitor of glutamine metabolism, led to the inhibition of pro-inflammatory cytokine production. Epigenetic assessment of histone methylation revealed increased trimethylation of H3K4 in the mTOR promoters, but also of the glycolysis-related enzymes hexokinase 2 and the platelet phosphofructokinase isoform. In addition, trimethylation of lysine 9 in histone 3 (H3K9) was observed, suggesting transcriptional repression. This involved mTOR and the above enzymes, as well as the glutamine metabolism enzymes, glutaminase and glutamate dehydrogenase. The authors also demonstrated increased phosphorylation of ribosomal protein S6 kinase 1 (S6K1), which is activated through mTORC1, thereby promoting mRNA translation. An analysis was also carried out with human, healthy volunteers who were given metformin for five days, after which monocytes were collected from them, and trained immunity was induced using BCG. The inhibitor suppressed the cytokine response and glycolysis, as evidenced by reduced lactate production [33]. Taken as a whole, the studies presented here demonstrated the importance of PI3K/Akt-mTOR pathways, glycolysis and glutamine metabolism in the development and maintenance of the trained immunity process.

Another metabolic pathway involved in trained immunity is the cholesterol synthesis/mevalonate pathway. Bekkering et al. [34] conducted a study using human monocytes stimulated with both β-glucan and BCG. They showed increased activity of the cholesterol synthesis pathway. Two inhibitors were also used: fluvastatin (a 3-hydroxy-3-methyl-glutaryl-coenzyme A reductase (HMG-CoA) inhibitor) and Zaragozic acid A (a squalene synthesis inhibitor), the first of which led to a reduction in the synthesis of pro-inflammatory cytokines, while the second did not. Cholesterol synthesis alone may not be necessary for the development of trained immunity. Interestingly, 6-fluoromevalonate, an inhibitor of mevalonate pyrophosphate decarboxylase, enhanced the inflammatory response in the monocytes tested. Mevalonate pyrophosphate decarboxylase catalyses the formation of isopentenyl pyrophosphate from mevalonate pyrophosphate, so its blockade leads to the accumulation of mevalonate, which appears to be crucial here. When added to monocyte cultures, mevalonate restored the trained immunity previously blocked by fluvastatin. Mevalonate also enhanced H3K4 trimethylation in the promoter region of IL-6 and TNF-α genes [34]. It is worth mentioning that IL-6 and TNF-α are the primary cytokines secreted in response to an infectious agent, but in the context of SARS-CoV-2 infection, they have an important role in the development of excessive inflammation, known as a cytokine storm. This can lead to deterioration of the general condition, respiratory distress and death [35]. Mevalonate is known to affect the insulin-like growth factor-1 receptor (IGF-1R) [36], so the authors also assessed the effect of an IGF-1R inhibitor (human antibody) on the trained immunity stimulated by β-glucan. A similar attenuating impact on the process was obtained. In addition, the authors collected material from three patients with the hyper-IgD syndrome (OMIM #260920), an autoinflammatory disease whose molecular basis is mevalonate kinase deficiency [37,38]. The production of the pro-inflammatory cytokines (IL-1β, IL-6 and TNF-α) was significantly higher in harvested monocytes than in healthy controls [34]. Mevalonate is another important metabolite that can mediate the process of trained immunity. This knowledge offers excellent potential for application in clinical practice but needs to be further explored in detail.

Cirovic et al. [39] approached the topic of BCG-induced trained immunity from bone marrow progenitor cells. The authors administered BCG vaccination to 15 healthy, previously unvaccinated study participants, followed by evaluation of cells from blood and bone marrow biopsies two weeks and three months after vaccination. BCG led to increased production of pro-inflammatory cytokines following monocyte restimulation by *Candida albicans*. However, more interestingly, transcriptomic analysis of haematopoietic stem and progenitor cells revealed increased activity of specific genes and signalling pathways. Of these, two genes, *HNF1A* and *HNF1B*, encoding Hepatocyte nuclear factor-1α (HNF-1α) and Hepatocyte nuclear factor-1β (HNF-1β), respectively, appeared to be the most significant. These molecules, in addition to hepatocytes, are also secreted by bone marrow cells. The target genes of the above, *SERPINA1* and *SERPINA10*, were also up-regulated. The protein product of *SERPINA1* is an antiprotease, α1-antitrypsin, whose levels were also increased three months after BCG vaccination. In addition, the authors assessed single nucleotide polymorphisms (SNPs) in the HNF1A gene showing variants that influenced TNFα production to a greater or lesser extent in BCG-stimulated trained immunity. The study pointed to the broader importance of the trained immunity process involving not only peripheral cells but also bone marrow progenitor cells.

Another factor affecting the phenomenon of trained immunity is the type of diet consumed. Christ et al. [40] conducted a study on mice assessing the effect of a Western-type diet. The first conclusion from the study was that the Western diet caused transient hypercholesterolaemia and an inflammatory response. An analysis was also performed on monocytes, exposing them to an oxidised form of low-density lipoprotein (oxLDL), which, as for BCG, can generate trained immunity. Monocytes incubated with oxLDL after restimulation with lipopolysaccharide (LPS) showed an enhanced response capability compared with cells incubated with a normal culture medium. In addition, the authors performed SNP analysis, finding an SNP in the region encoding, among others, the IL-1 receptor antagonist (IL-1ra), which may have influenced the induction of trained immunity by oxLDL. After the administration of recombinant IL-1ra, the monocyte response and production of pro-inflammatory cytokines were reduced. This highlighted the role of IL-1 in the process in question.

To complement the knowledge of the above molecular studies, it is worth citing the work of Kong et al. [41] based on transcriptomic analysis. The authors performed transcriptomic evaluation (RNA sequencing) of a single monocyte cell before and after inoculation with BCG and after restimulation with bacterial LPS. In this study, BCG, in turn, inhibited the systemic inflammatory response, but genes associated with the trained immunity process were also identified, including chemokine genes Chemokine (C-C motif) ligand 3 (CCL3) and Chemokine (C-C motif) ligand 4 (CCL4) [41]. 

In conclusion, the large number of studies carried out on different models showed that multiple signalling pathways and epigenetic modifications induced by BCG are responsible for the trained immunity effect (Table 1). We will probably also learn further about the trained immunity process through data obtained during the coronavirus disease pandemic 2019 (COVID-19).

## 3. SARS-CoV-2 and Trained Immunity

A new coronavirus causing respiratory illness was first reported in December 2019 in the city of Wuhan, China. COVID-19 disease presents mainly with fever and cough. Still, it is also associated with acute respiratory failure and complications from multiple organs, including the heart, kidneys, gastrointestinal tract and central nervous system [42,43]. The primary receptor through which the virus enters target cells is the Angiotensin-converting enzyme 2 [44]. During the COVID-19 pandemic, the hypothesis, partly supported by observations, was suggested that countries without widespread BCG prophylaxis experienced an increased incidence of the disease [45]. Using knowledge of the trained immunity phenomenon and the experience of previous observational studies, many authors have attempted to understand the effect of BCG vaccination on the course and severity of SARS-CoV-2 infection [46]. Various studies have been conducted on different populations and with different scientific methodologies.

A notable proportion of trials included observational studies and controlled clinical trials. Weng et al. [47] studied a group of 120 adult patients with COVID-19, of whom 82 patients (68.3%) were vaccinated with BCG, and this group had a lower risk of hospitalisation (*p* = 0.019). Khanum et al. [48] conducted an observational study on adults with a positive polymerase chain reaction (PCR) test for SARS-CoV-2. The study evaluated 103 patients with COVID-19, 64 of whom were vaccinated against TB. The authors showed no difference in the severity of COVID-19, whereas they demonstrated reduced mortality in the BCG-vaccinated group.

In a study by Amirlak et al. [49], 71 patients were revaccinated from a group of 280 healthcare workers previously vaccinated with BCG. The authors found 18 cases of SARS-CoV-2 infection among hospital staff during a 3-month follow-up period. Interestingly, all patients were in the group that had not received an additional dose of the BCG vaccine. In a multicentre study, Torun et al. [50] analysed 465 medical professionals with SARS-CoV-2 infection for contact with *Mycobacterium tuberculosis*. The authors showed, in turn, that the hospitalised study participants had a history of direct contact with tuberculosis. However, only one person in the hospitalised group (217 participants) died from COVID-19. It is possible that the trained immunity process may have influenced the reduced mortality in this study. Still, the results are inconclusive, and the study’s observational nature was a significant limitation.

Rivas et al. [51] analysed more than 6000 healthcare workers for BCG vaccination; 29.6% were vaccinated, and 68.9% were not. The reporting of COVID-19 disease symptoms was significantly lower in the BCG-vaccinated group compared with the unvaccinated group. The authors additionally analysed the study participants for other microbial vaccinations (*Neisseria meningitidis*, *Streptococcus pneumoniae* and influenza). In this case, no significant results were obtained. Tsilika et al. [52] conducted a randomised, double-blind clinical trial on a group of 516 elderly patients. Of this group, 301 received BCG or a placebo. Re-vaccination with BCG reduced the risk of SARS-CoV-2 infection to 68%.

Dos Anjos et al. [53] conducted a single-centre study among healthcare workers with BCG revaccination. The authors showed a reduction in the incidence of COVID-19 in the vaccinated group, but statistical significance was not achieved. Jalalizadeh et al. [54] conducted a clinical trial on a group of 378 adult patients recovering from COVID-19 disease. Participants vaccinated with BCG had a more marked return of smell and taste at six weeks of follow-up compared with the placebo group. Dionato et al. [55] evaluated the safety of BCG revaccination in patients in recovery from COVID-19, finding no adverse effects.

Similar results evaluating the effect of BCG on the course of SARS-CoV-2 infection from several different approaches (medical interventions, absenteeism from work, type I diabetes individuals) have been reported by other authors [56,57,58]. Additionally, our team conducted a double-blind, placebo-controlled clinical trial [41,59]. We assessed the effect of BCG re-vaccination on COVID-19 among medical professionals but did not obtain statistical significance between study groups. 

Despite an increasing number of studies exploring this area, no work has yet been published that focuses on the molecular aspect of the effect of the SARS-CoV-2 virus on the trained immunity process. We also do not know to what extent this pathogen can influence the phenotype of BCG-induced innate immunity cells. Ahmed et al. [60] performed a database analysis of epigenomic and transcriptomic changes in monocytes after BCG vaccination. They showed that some genes were up-regulated in monocytes, including TLR receptor pathway genes and interferon-inducible genes, e.g., C-X-C motif chemokine ligand 10 (CXCL10). This may indicate the activation of a non-specific antiviral response so that the reaction against SARS-CoV-2 may be more effective. However, it is likely that this is not the only mechanism that may affect the antiviral response. Clearly, this topic requires further research. The summary of the current observational and clinical trials on BCG-COVID-19 relation is provided in Table 2.

## 4. Challenges and Perspective

Based on the above studies, one may be inclined to believe that the BCG vaccine may have applicability in the protection or therapy of COVID-19 disease. Despite the specific vaccines against the SARS-CoV-2 virus that have been produced, this may be an additional tool. During SARS-CoV-2 infection, there is a reduced interferon response so that the virus can replicate more efficiently. Blanco-Melo et al. [61] performed a transcriptomic evaluation of human bronchial epithelial cells under in vitro culture conditions and in an animal ferret model. In both cases, the production of type I and III interferons were significantly decreased after SARS-CoV-2 infection. In addition, the authors analysed lung samples from individuals who died from COVID-19, showing increased expression of chemokine genes without up-regulation of interferon transcription. The use of BCG can reverse this effect [61]. This is particularly important in the context of developing countries, where the availability, as well as storage capabilities, of mRNA-based vaccines, are limited. 

Despite its promising nature, this idea is not free of limitations and challenges. Firstly, the BCG vaccine contains a live strain of *Mycobacterium*, and, just as for other live vaccines, it can be dangerous in specific clinical cases, leading to generalised infection. These include primary immunodeficiencies, high doses of immunosuppressive drugs or human immunodeficiency virus (HIV) infection. It is not precisely known how long the trained immunity effect persists after BCG vaccination, but some studies indicate that it may be 3 to 5 years [62]. This means that such use would need to be repeated. 

On the other hand, the BCG vaccine may stimulate a Th1 and Th17 type inflammatory response. This type of T-cell activation may lead to increased inflammatory response, cytokine storm, and worsen the COVID-19 clinical course [63,64,65]. However, it is difficult to say unequivocally whether such an effect will occur.

The only route of administration of BCG used in humans is intradermal administration. Studies in non-human primates have shown greater efficacy of other drug administration routes, including intravenous or nasal delivery, which may be important for infections with the virus affecting the respiratory system [46,63,66,67].

Despite several clinical studies, we do not yet have a comprehensive understanding of the effect of BCG on COVID-19 itself, and knowledge is constantly being improved. Many factors may influence the vaccine’s process and applicability, including age, membership of a specific population, geographical factors, diet, medications used, research methodology or regulatory aspects.

**Figure 1:**
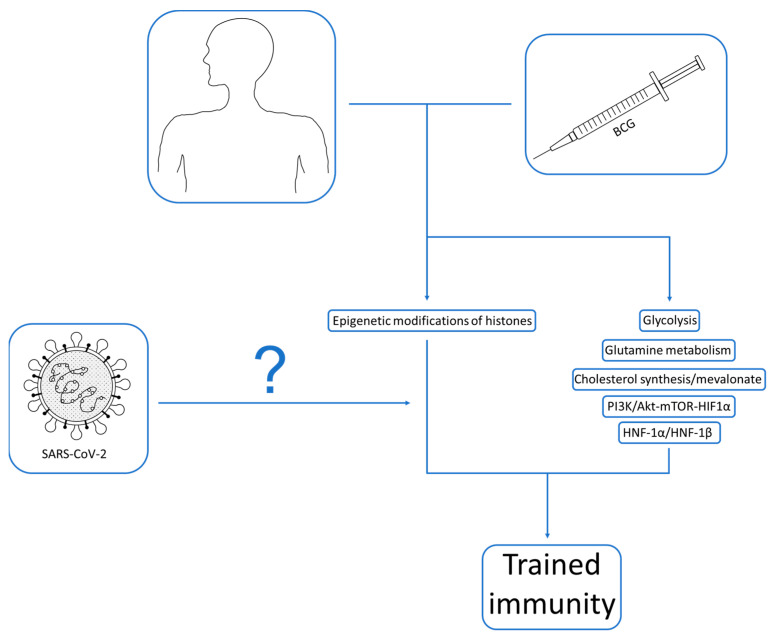
Schematic representation of the interactions between BCG, SARS-CoV-2 and trained immunity.

Indeed, the question of the effect of BCG vaccination on SARS-CoV-2 infection and the modulation of the trained immunity process by this virus requires further research. From the perspective of globalisation and the 2020 pandemic, it seems most relevant to investigate the safety of BCG in the above usage. An adequate follow-up to detect adverse reactions and potential risks is necessary. It is also essential to gain an in-depth understanding of the molecular mechanisms responsible for the above interaction. With such data, we will be able to create an additional protection tool against various infectious agents, as well as provide new insights into a vaccine that, despite 100 years of history, is still not fully understood. Additionally, it is worth mentioning the comprehensive review by Brueggeman et al. [68], which outlines the process of trained immunity in general, the main stimulating agents, as well as a description of monocytes and NK cells in this phenomenon. The authors also referred to COVID-19 and the possibility of using current knowledge in further management. A summary of the challenges of the trained immunity process, BCG and SARS-CoV-2 are presented in Figure 1.

## Figures and Tables

**Table 1:** Summary of studies on the molecular mechanisms of trained immunity.

Type of Study	Organism	Stimulating Factor	Results	Reference
Cytokine and epigenetic analysis	Human monocytes, mouse model	BCG, *Candida albicans*, *Staphylococcus aureus*	Increased levels of proinflammatory cytokines; epigenetic changes in promoters of proinflammatory genes and TLR4	[26]
Identification of metabolic pathways	Human monocytes, mouse model	β-glucan	Activation of glycolysis and mTOR pathway	[30]
Identification of metabolic pathways	Human monocytes, mouse model	BCG	Activation of glycolysis, mTOR pathway, and glutamine metabolism	[33]
Identification of metabolic pathways	Human monocytes, mouse model	BCG, β-glucan	Activation of mevalonate pathway	[34]
Identification of signalling pathways	Human monocytes and bone marrow cells	BCG, *Candida albicans*	Activation of HNF-1α/HNF-1β	[39]
Evaluation of the impact of diet	Human monocytes, mouse model	oxLDL, LPS	Western-type diet increased inflammatory response	[40]

Abbreviations: BCG—Bacillus Calmette–Guérin; HNF-1α—Hepatocyte nuclear factor-1α; HNF-1β—Hepatocyte nuclear factor-1β; LPS—Lipopolysaccharide; mTOR—mammalian target of rapamycin; oxLDL—oxidised form of low-density lipoprotein; TLR4—Toll-like receptor type 4.

**Table 2:** Summary of studies of the effect of BCG on SARS-CoV-2 infection.

Type of Study	Study Population	Country	Results	Statistical Significance	Reference
Observational study	120 adult COVID-19 patients of a federal health care centre	United States of America	The BCG-vaccinated group had a lower risk of hospitalisation compared with the unvaccinated group.	Significant	[47]
Observational study	103 SARS-CoV-2 PCR positive adult patients	Pakistan	No significant differences in the course of COVID-19. Lower mortality in the BCG-vaccinated group.	Depending on the parameter	[48]
Observational study	465 healthcare workers with SARS-CoV-2 infection	Turkey	Hospitalised patients had a history of direct contact with tuberculosis. Lower mortality in this group.	Significant	[50]
Observational study	6201 healthcare workers	United States of America	COVID-19 disease symptoms was significantly lower in the BCG-vaccinated group.	Significant	[51]
Cohort study with revaccination	280 healthcare workers previously vaccinated with BCG	United Arab Emirates	SARS-CoV-2 infection occurred only in the non-revaccinated group.	Significant	[49]
Clinical trial	516 elderly patients	Greece	Reduced risk of SARS-CoV-2 infection.	Significant	[52]
Clinical trial	138 healthcare workers	Brazil	Reduction in the incidence of COVID-19 in the vaccinated group.	Insignificant	[53]
Clinical trial	378 adult patients recovering from COVID-19	Brazil	BCG-vaccinated participants had increased return of smell and taste at six weeks of follow-up. No adverse effects of BCG revaccination in adult patients.	Significant	[54,55]
Clinical trial	2014 elderly patients	The Netherlands	No differences in incidence of infections, including COVID-19. Reduced number of days of dyspnoea in the BCG-vaccinated group.	Depending on the parameter	[56]
Clinical trial	1511 healthcare workers	The Netherlands	No difference in the number of days of absenteeism from work.	Insignificant	[57]
Clinical trial	144 adult patients with type I diabetes	United States of America	Lower incidence of COVID-19 in the BCG-vaccinated group.	Significant	[58]
Clinical trial	717 healthcare workers	Poland	No difference in incidence of COVID-19 between the vaccinated and unvaccinated group.	Insignificant	[41,59]

Abbreviations: BCG—Bacillus Calmette–Guérin; COVID-19—coronavirus disease 2019; PCR—Polymerase chain reaction; SARS-CoV-2—severe acute respiratory syndrome coronavirus 2.

## Data Availability

Not applicable.

## Abbreviations

ADP	Adenosine diphosphate
AMP	Adenosine monophosphate
AMPK	AMP-activated protein kinase
APCs	Antigen presenting cells
BCG	Bacillus Calmette–Guérin
BPTES	Bis-2-(5-phenylacetamido-1,3,4-thiadiazol-2-yl)ethyl sulfide
CCL3	Chemokine (C-C motif) ligand 3
CCL4	Chemokine (C-C motif) ligand 4
CD4^+^	Cluster of differentiation 4
CD8^+^	Cluster of differentiation 8
CD11b	Cluster of differentiation 11b
CD14	Cluster of differentiation 14
CLRs	C-type lectin receptors
COVID-19	Coronavirus disease 2019
CXCL10	C-X-C motif chemokine ligand 10
DAMPs	Danger-associated molecular patterns
DC	Dendritic cell
DNA	Deoxyribonucleic acid
DTP	Diphtheria, tetanus, pertussis
GMP	Guanosine monophosphate
H3K4	Histone 3 lysine 4
H3K9	Histone 3 lysine 9
HIF1α	Hypoxia-inducible factor 1 α
HIV	Human immunodeficiency virus
HNF-1α	Hepatocyte nuclear factor-1α
HNF-1β	Hepatocyte nuclear factor-1β
HMG-CoA	3-hydroxy-3-methyl-glutaryl-coenzyme A
IFN-γ	Interferon γ
IGF-1R	Insulin-like growth factor-1 receptor
IgG	Immunoglobulin G
IL-1β	Interleukin 1-β
IL-1ra	Interleukin 1 receptor antagonist
IL-6	Interleukin 6
LPS	Lipopolysaccharide
MHC	Major histocompatibility complex
mRNA	Messenger ribonucleic acid
mTOR	Mammalian target of rapamycin
mTORC1	mTOR complex 1
mTORC2	mTOR complex 2
NAD^+^	Nicotinamide adenine dinucleotide oxidised form
NADH	Nicotinamide adenine dinucleotide reduced form
NLRs	Nucleotide-binding oligomerisation domain-like receptors
PAMPs	Pathogen associated molecular patterns
PCR	Polymerase chain reaction
PI3K/Akt	Phosphoinositide 3-kinase/protein kinase B
PRRs	Pattern recognition receptors
RLRs	Retinoic acid-inducible gene-I-like receptors
S6K1	Ribosomal protein S6 kinase 1
SARS-CoV-2	Severe acute respiratory syndrome coronavirus 2
SCID	Severe combined immunodeficiency
TB	Tuberculosis
Th1	T helper type 1
Th17	T helper type 17
TLR4	Toll like receptor 4
TLRs	Toll-like receptors
TNF-α	Tumour necrosis factor α
